# S1PR2 participates in intestinal injury in severe acute pancreatitis by regulating macrophage pyroptosis

**DOI:** 10.3389/fimmu.2024.1405622

**Published:** 2024-05-17

**Authors:** Tianjiao Lin, Mengyuan Peng, Qingyun Zhu, Xinting Pan

**Affiliations:** Emergency Intensive Care Unit, The Affiliated Hospital of Qingdao University, Qingdao, Shandong, China

**Keywords:** severe acute pancreatitis, S1PR2, pyroptosis, intestinal injury, macrophage - cell

## Abstract

**Background:**

Severe acute pancreatitis (SAP) is an inflammatory disorder affecting the gastrointestinal system. Intestinal injury plays an important role in the treatment of severe acute pancreatitis. In this study, we mainly investigated the role of S1PR2 in regulating macrophage pyroptosis in the intestinal injury of severe acute pancreatitis.

**Methods:**

The SAP model was constructed using cerulein and lipopolysaccharide, and the expression of S1PR2 was inhibited by JTE-013 to detect the degree of pancreatitis and intestinal tissue damage in mice. Meanwhile, the level of pyroptosis-related protein was detected by western blot, the level of related mRNA was detected by PCR, and the level of serum inflammatory factors was detected by ELISA. *In vitro* experiments, LPS+ATP was used to construct the pyroptosis model of THP-1. After knockdown and overexpression of S1PR2, the pyroptosis proteins level was detected by western blot, the related mRNA level was detected by PCR, and the level of cell supernatant inflammatory factors were detected by ELISA. A rescue experiment was used to verify the sufficient necessity of the RhoA/ROCK pathway in S1PR2-induced pyroptosis. Meanwhile, THP-1 and FHC were co-cultured to verify that cytokines released by THP-1 after damage could regulate FHC damage.

**Results:**

Our results demonstrated that JTE-013 effectively attenuated intestinal injury and inflammation in mice with SAP. Furthermore, we observed a significant reduction in the expression of pyroptosis-related proteins within the intestinal tissue of SAP mice upon treatment with JTE-013. We confirmed the involvement of S1PR2 in THP-1 cell pyroptosis *in vitro*. Specifically, activation of S1PR2 triggered pyroptosis in THP-1 cells through the RhoA/ROCK signaling pathway. Moreover, it was observed that inflammatory factors released during THP-1 cell pyroptosis exerted an impact on cohesin expression in FHC cells.

**Conclusion:**

The involvement of S1PR2 in SAP-induced intestinal mucosal injury may be attributed to its regulation of macrophage pyroptosis.

## 1 Introduction

Acute pancreatitis (AP) is a pancreatic inflammatory disease characterized by local acinar damage and systemic inflammatory response. The global annual incidence is about 34/100,000 people, and the trend is increasing year by year (1). Pancreatic necrosis and intestinal function impairment caused by early acinar cell death are still important causes of severe disease in patients (2, 3). Severe acute pancreatitis (SAP) is a common critical condition primarily caused by pancreatic injury and systemic organ damage. It is accompanied by early-stage macrophage infiltration and release of numerous pro-inflammatory factors into the bloodstream, leading to systemic inflammation. In cases of SAP occurrence, the intestinal tract, as a distant organ, may experience mucosal ischemia and hypoxia while also disrupting the normal intestinal flora, thereby exacerbating the disease.

G protein-coupled receptors (GPCRs) are a ubiquitous type of membrane receptor that play crucial roles in cell signal transduction, regulating various physiological processes such as chemoreception, immune regulation, neurotransmission and endocrine regulation. S1PR2 is a highly expressed subtype of GPCR primarily found in vascular smooth muscle cells, heart, liver, kidney, spleen, lung and brain. There are five subtypes of S1PRs: S1PR1-5 (4). The G protein family can be categorized into four distinct families: Gq/11, G12/13, Gs, and Gi/o. The S1PR2 and S1PR3 receptors possess the capability to interact with G12/13, Gq/11, and Gi/o proteins, whereas the S1PR1 and S1PR5 receptors demonstrate a preference for binding specifically to Gi/o proteins. Previous research has demonstrated that activation of different types of cells by S1PRs leads to diverse biological effects; for instance, S1PR2 is involved in the migration and invasion processes of multiple myeloma cells as well as inhibiting proliferation in SKOV3 cells during allergic asthma or epithelial ovarian cancer (5). However, it is elusive that the precise mechanism underlying the involvement of S1PR2 in intestinal injury induced by SAP. In this study, our objective is to elucidate the mechanistic involvement of S1PR2 in SAP pathogenesis, thereby offering novel insights for therapeutic intervention.

The GSDMD was identified through a comprehensive whole genome editing screening conducted by Academician Shao Feng’s team (6), and the Gasdermin family proteins, exemplified by GSDMD, have been progressively validated as the ultimate effector proteins of pyroptosis. Pyroptosis has emerged as a prominent research focus in recent years, and the investigation of pyroptosis holds significant pathophysiological implications for disease management. Currently, it has been found that pyroptosis plays a key role in inflammatory diseases, oncologic diseases, immune diseases (7, 8). The severity of intestinal injury in SAP is closely associated with prognosis, and pyroptosis represents a form of cellular demise. However, the precise role of pyroptosis in intestinal injury induced by SAP remains incompletely elucidated.

In this paper, we found that inhibition of S1PR2 expression by JTE-013 can alleviate SAP in mice. In addition, we observed a simultaneous decrease in the expression of pyroptosis-related proteins in mice with JTE-013. Moreover, it was verified that S1PR2 was involved in macrophage pyroptosis and further affected intestinal cells *in vitro*. Therefore, we mainly discussed the role of S1PR2 in regulating macrophage pyroptosis to participate in the intestinal mucosal barrier function of SAP.

## 2 Materials and methods

### 2.1 Animal experiments

The animal experiments were conducted in accordance with the guidelines provided by the Animal Care and Welfare Committee of the Affiliated Hospital of Qingdao University (AHQU-MAL20220708). Twenty-four SPF-grade male C57BL/6 mice, weighing 20 ~ 25g and meeting SPF-grade standards, were raised by the Animal Experimental Center of Huangdao Hospital, Affiliated Hospital of Qingdao University. The mice were divided into Control (CON) group, SAP model group, and JTE-013 group by random number table method, with 8 mice in each group.

The C57BL/6 mice were subjected to an 18-hour fasting period prior to the experiment, while being provided with unrestricted access to water. The SAP model group was given intraperitoneally 50 μg/kg cerulein (Meilunbio,Dalian,China), once an hour for 6 consecutive times, 10 mg/kg lipopolysaccharide(Meilunbio) was given after the last injection of cerulein, the CON group was given the same dose of normal saline, the JTE-013 group received the same preliminary treatment as SAP group, JTE-013 4 mg/kg for the last time (9).

Whole blood was anesthetized 12 h after modeling, pancreas and ileal tissues were fixed in formalin solution, and part of the ileal tissue was stored at -80°C for later use.

### 2.2 Cell culture, transfection and treatment

The human mononuclear macrophage THP-1 cells were cultured in RPMI 1640 complete medium (Procell, Wuhan, China) supplemented with 10% fetal bovine serum (Procell) and 0.05 mmol/L β-mercaptoethanol. Upon reaching a cell density of 80%-90%, the culture was passaged. Following passage, the THP-1 cells were incubated at 37°C in a 5% CO_2_ cell incubator to induce adhesion using culture medium containing 100 ng/ml Phorbol12-myristate13-acetate(MedChenExpress, New Jersey, United States) for a duration of 72 hours. Subsequently, the culture medium was replaced, and the THP-1 cells were further cultured for subsequent experiments.

The human intestinal epithelial FHC cells were cultured in a complete medium of DMEM (Procell) supplemented with 10% fetal bovine serum. Upon reaching a cell density of 80%-90%, the cells were digested using pancreatic enzyme, followed by centrifugation and subsequent passage at a ratio of 1:3. After passage, the cells were incubated in a cell culture chamber maintained at 37°C with 5% CO_2_ for further experimental procedures.

The THP-1 cell line was divided into four experimental groups: NC, LPS+ATP, LPS+ATP +siRNA, and LPS+ATP +shDNA group. The adherent THP-1 cells were cultured in antibiotic-free medium. The S1PR2 overexpression plasmid (Genechem, Shanghai, China) was transfected using Advanced DNA RNA Transfection Reagent (ZETALife, SanFrancisco, United States) while the S1PR2 siRNA (GenePharma, Shanghai, China) was transfected using Lipofectamine 2000 (Invitrogen, California, UnitedStates)(Sense: GUAGCCAAUACCUUGCUCUTTantisense: AGAGCAAGGUAUUGGCUACTT). Subsequently, the THP-1 cells were stimulated with 1 μg/ml of LPS for 24 hours followed by induction with 5 mmol/L ATP for 20 minutes.

The THP-1 cell line was inoculated into the lower chamber of a 6-well plate, while FHC cells were inoculated into another chamber of the same plate. Once reaching approximately 90% confluency, the chambers were transferred to the 6-well plate and co-cultured with THP-1 cells. Following incubation, cells within the chambers were harvested for subsequent experimental procedures.

### 2.3 Enzyme-linked immunosorbent assay

Serum of mice and cell supernatant were initially obtained in accordance with the instructions. The procedure followed the instructions provided with the ELISA kit (ElabScience, Wuhan, China), and the optical density (OD) value was measured and analyzed using a microplate reader.

### 2.4 Hematoxylin and eosin staining

We collected pancreatic and intestinal tissues from mice, which were subsequently fixed in a formalin solution. The tissues were then embedded in paraffin using conventional methods and stained with hematoxylin-eosin (HE). Subsequently, the sections were observed under a light microscope.

### 2.5 Immunohistochemical staining

The paraffin sections underwent dewaxing and hydration procedures. Then 3% H_2_O_2_ was used to inhibit endogenous peroxidase activity, and sections were incubated with the primary antibodies severally including ZO-1 antibody (1:200, Abcam, Cambridge, United Kingdom), Occludin (1:200, Abcam), Claudin-1 (1:200, Abcam), and S1PR2 antibody (1:200 Affinity Biosciences, Jiangsu, China) at 4°C. This was followed by a 20-minute incubation with HRP-anti-rabbit Ig-G (Absin, Wuhan, China). The sections were subjected to DAB and hematoxylin staining, followed by microscopic examination for result visualization.

### 2.6 Quantitative real-time polymerase chain reaction

The RNAeasy (Vazyme, Nanjing, China) was used to extract tissue or intracellular RNA, and the concentration and purity were detected by the ultraviolet absorption method, then cDNA was synthesized by reverse transcription according to the kit requirements (Vazyme). The primer sequences (Sangon biotech, Shanghai, China) are shown in the table (**Tables 1**, **2**).

**Table 1:** Primer sequences for the mouse gene.

Primer	Sequence
S1PR2	F’:CATCTTACTGGCTATCGTGGCTCTG
	R’:GCTTCTGAGGACCAGCAACATC
NLRP3	F’:CCAGACCTCCAAGACCACTACG
	R’:CAGAGAAGAGATGCTCCTCAATGC
Caspase1	F’:ATACAACCACTCGTACACGTCTTGC
	R’:TCCTCCAGCAGCAACTTCATTTCTC
Caspase11	F’:GGCTACGATGTGGTGGTGAAAGAG
	R’:ATGTGCTGTCTGATGTCTGGTGTTC
GSDMD	F’:ACTGAGGTCCACAGCCAAGAGG
	R’:GCCACTCGGAATGCCAGGATG
Occludin	F’:GGCGGCTATGGAGGCTATGG
	R’:CTAAGGAAGCGATGAAGCAGAAGG
ZO-1	F’:AGCAGTGGAAGAAGTTACAGTTGAG
	R’:TAGGCAGAGCACCATCAGAAGG
Claudin-1	F’:CCTGGCTTCTCTGGGATGGAT
	R’:CTGAGCGGTCACGATGTTGTC

**Table 2:** Primer sequences for THP-1.

Primer	Sequence
S1PR2	F’:GCCTTCATCGTCATGCTCTGTTG
	R’:CAGGTACATTGCCGAGTGGAAC
NLRP3	F’:CGTGAGTCCCATTAAGATGGAGT
	R’:CCCGACAGTGGATATAGAACAGA
Caspase1	F’- AAAAGCCATGGCCGACAAG
	R’-TCTTCCTTGTTCAGCACCCT
Caspase4	F’-TTCCCTATGGCAGAAGGCAAC
	R’-TGCCATGACCCGAACTTTGT
GSDMD	F’:TGCCTCCACAACTTCCTGACAG
	R’:GTCTCCACCTCTGCCCGTAG

### 2.7 Immunofluorescence staining

Pancreatic sections were incubated with primary antibodies against amylase (1:100, Affinity Biosciences) and S1PR2 (1:100, Affinity Biosciences) overnight at 4°C. The sections were subsequently incubated with fluorescent secondary antibodies and counterstained with DAPI. Images were acquired by Olympus microscope.

### 2.8 Western blot

A total of 20mg of mouse intestinal tissue or cells in a 6-well plate were collected. Subsequently, the samples were lysed using RIPA buffer to extract proteins. For electrophoresis and membrane transfer, 10μg of protein was utilized. Following milk blocking, the membrane was incubated overnight at 4°C on a shaker with different primary antibodies separately, including S1PR2 (1:500,Affinity Biosciences), Caspase1 (1:1000, Affinity Biosciences), Caspase11 (1:1000, Affinity Biosciences), GSDMD (1:1000, Affinity Biosciences), NLRP3 (1:1000, Abcam, Cambridge, United Kingdom), ZO-1 (1:1000,Abcam), Occludin (1:1000,Abcam), ROCK1 (1:1000, Abcam), RhoA (1:5000, Abcam), β-Tubulin (1:1000, BOSTER, Wuhan, China), β-actin (1:1000, BOSTER). After thorough membrane washing, secondary antibodies (1:5000, BOSTER) were applied and finally visualized using a visualizer. The obtained results were processed utilizing ImageJ.

### 2.9 Statistic analysis

The statistical analysis was performed using GraphPad Prism 8.0.2 software. Measurement data were presented as mean ± standard deviation. Differences between groups were analyzed using one-way analysis of variance (ANOVA). Statistical significance was defined as *P<0.05*.

## 3 Results

### 3.1 JTE-013 alleviates pathological injury in SAP-induced mice

**Figure 1:**
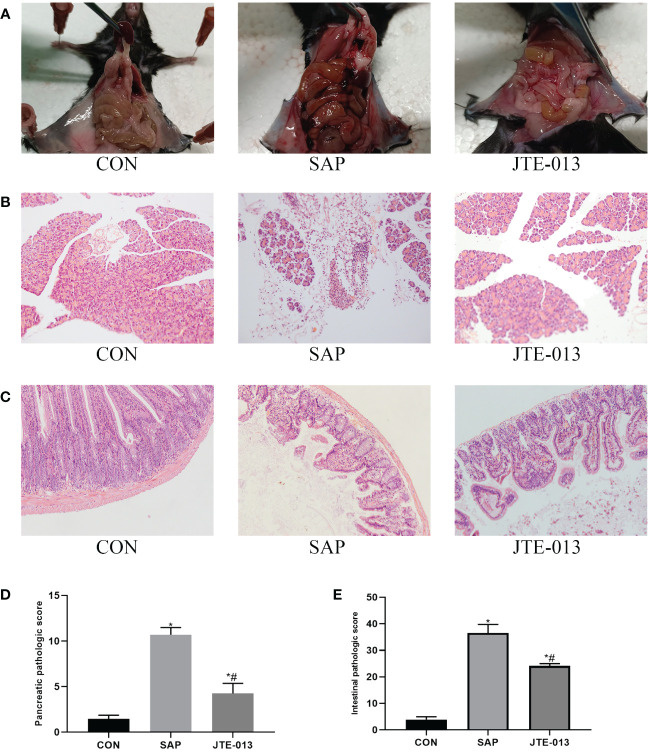
JTE-013 alleviates pancreatic and intestinal damage in mice with SAP. **(A)** The intestinal and pancreatic lesions of mice were observed. **(B)** Results of pancreatic pathological injury (HE*200). **(C)** Results of intestinal pathological injury (HE*200). **(D)** The score of pancreatic pathology. **(E)** The score of intestinal pathology. *P<0.05 VS.CON #P<0.05 VS.SAP.

Firstly, we investigated the effect of JTE-013 in attenuating pathological damage in mice with SAP. Following intraperitoneal injection of 4mg/kg reagent in the JTE-013 group, a significant reduction in pancreatic edema and ascites was observed compared to the SAP group (**Figure 1A**). HE revealed that infiltration of inflammatory cells in the pancreas was more severe in the SAP group than in the JTE-013 group, with a statistically significant difference according to the pancreatic pathology scoring criteria proposed by Schmidt (10) (**Figures 1B, D**). Furthermore, light microscopy analysis of intestinal HE samples showed increased intervillous space along with inflammatory cell infiltration and edema in the JTE-013 group. In contrast, the SAP group exhibited obvious destruction of intestinal mucosal villi accompanied by pronounced inflammatory cell infiltration and interstitial edema. According to Chiu’s grading criteria (11), there were significant differences in intestinal mucosal pathological scores among the groups (**Figures 1C, E**, *P<0.05*).

### 3.2 JTE-013 reduced inflammation in mice with SAP

**Figure 2:**
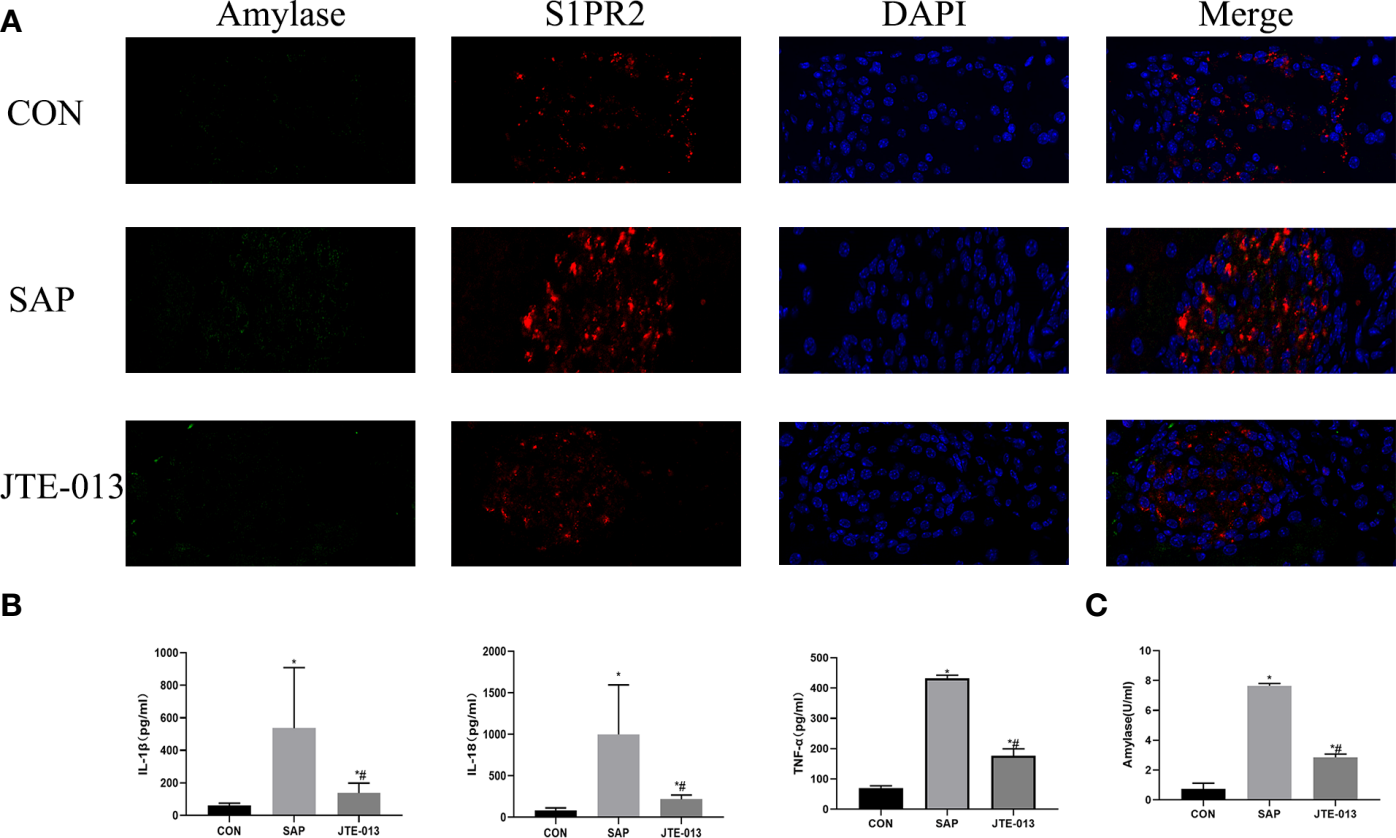
JTE-013 reduces pancreatic amylase levels and serum inflammation levels. **(A)** Results of pancreas S1PR2 and amylase detected by immunofluorescence double staining. **(B)** Serum levels of inflammatory cytokines IL-18, IL-1β, TNF-α detected by ELISA. **(C)** Serum amylase levels detected by colorimetric method *P<0.05 VS.CON #P<0.05 VS.SAP.

The levels of serum amylase and inflammatory cytokines, including IL-1β, IL-18, and TNF-α, were quantified. Our findings revealed significantly elevated concentrations of serum amylase and inflammatory factors in the SAP group compared to the CON group. Furthermore, the JTE-013 group exhibited higher levels of serum amylase and inflammatory factors than the CON group but lower than those observed in the SAP group (**Figures 2B, C**, *P<0.05*).

Then, the localized expression of S1PR2 and amylase was further examined by immunofluorescence staining. It showed that S1PR2 was located in the cell membrane and was red. The quantity of amylase in the SAP group was higher than that in the CON group, and the difference was statistically significant (*P<*0.05), the relative quantification of the JTE-013 group was higher than that of the CON group, and the difference was statistically significant (**Figure 2A**, *P<*0.05).

### 3.3 JTE-013 attenuated pyroptosis in intestinal tissues of mice with SAP

**Figure 3:**
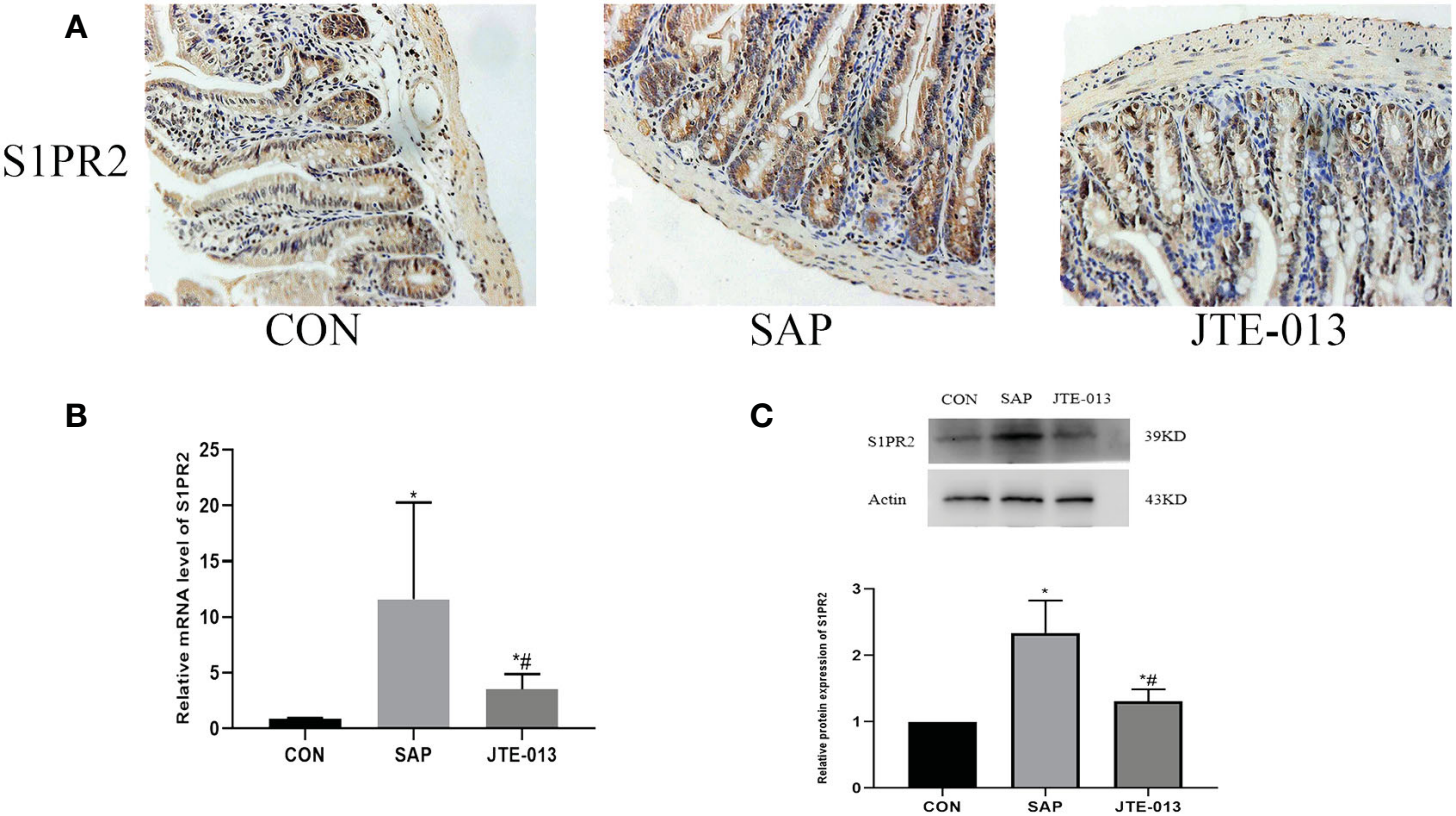
JTE-013 inhibited the expression of S1PR2 **(A)** The expression of intestinal S1PR2 protein was detected by immunohistochemical staining. (ordinary microscope*400). **(B)** The mRNA level of S1PR2 in intestinal tissues detected by PCR. **(C)** The S1PR2 protein level of intestinal tissue detected by western blot. *P<0.05 VS.CON #P<0.05 VS.SAP.

The inhibitory effect of JTE-013 on S1PR2 was assessed by immunohistochemistry, polymerase chain reaction, and Western blotting to determine the levels of S1PR2 in the midgut tissues of each experimental group. The results demonstrated a statistically significant difference between groups (**Figures 3A–C**, *P<0.05*).

**Figure 4:**
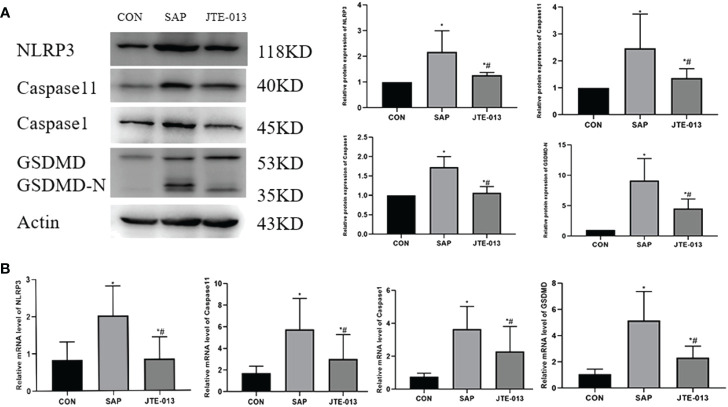
JTE-013 inhibited the expression of intestinal proteins related to pyroptosis in mice. **(A)** The pyroptosis protein level of intestinal tissue detected by western blot. **(B)** The mRNA level of pyroptosis in intestinal tissues detected by PCR. *P<0.05 VS.CON #P<0.05 VS.SAP.

Afterward, we proceeded to analyze the expression of pyroptosis proteins in the intestinal tissues of each experimental group of mice, encompassing NLRP3, Caspase1, Caspase11 and GSDMD-N. The expression of pyroptosis protein in the JTE-013 group was significantly lower compared to the SAP group, but higher than that in the CON group (**Figure 4A**). Similarly, the PCR results show that the NLRP3, Caspase1 and Caspase11, GSDMD mRNA level in the JTE-013 group was higher than that in the CON group and lower than that in the SAP group, and the difference was statistically significant (**Figure 4B**, *P<*0.05).

### 3.4 JTE-013 ameliorated intestinal tissue injury in mice with SAP

**Figure 5:**
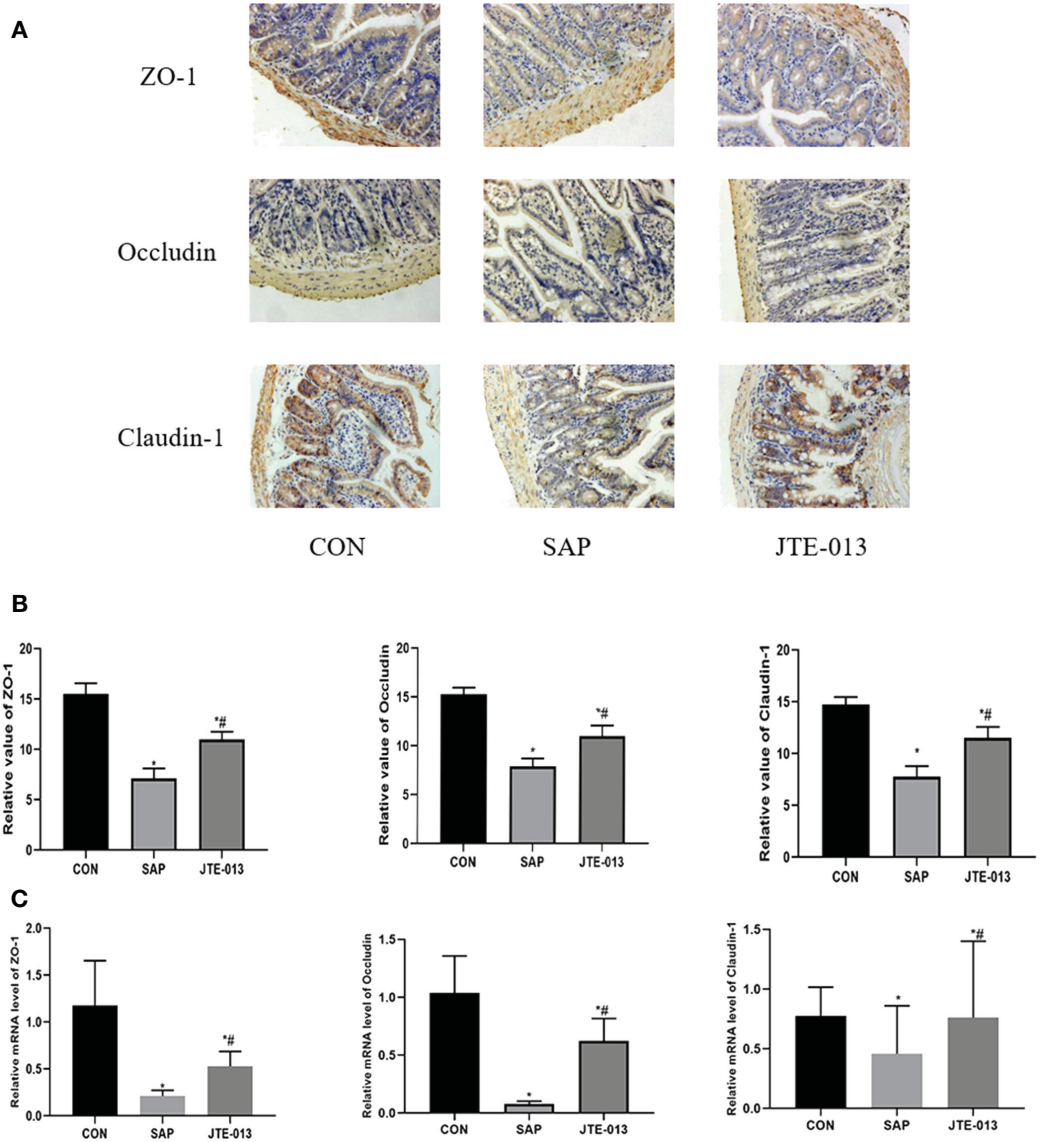
Expression of intestinal adhesion protein **(A)** The expression of intestinal adhesion protein was detected by immunohistochemical staining. (ordinary microscope*400). **(B)** The score of intestinal adhesion protein. **(C)** The mRNA level of ZO-1, Occludin and Claudin-1 in intestinal tissues detected by PCR. *P<0.05 VS.CON #P<0.05 VS.SAP.

The expression of adhesion protein was investigated through immunohistochemistry analysis, we found that in the CON group, Occludin, ZO-1, and Claudin-1 proteins were observed to be distributed along the intestinal mucosal epithelial cells with strong brown linear signals. Although the location of Occludin, ZO-1 and Claudin-1 in the JTE-013 group was not significantly different from that in the CON group, there was a noticeable weakening of brown positive signal. The number of positive cells decreased significantly in both SAP and JTE-013 groups. The relative quantitative difference of protein expression among these three groups was statistically significant (**Figures 5A, B**).

At the same time, we also detected the level of mRNA in intestinal tissues. The results showed that the Occludin, ZO-1, and Claudin-1 mRNA in the SAP group was lower than that in the CON group, and the Occludin, ZO-1, and Claudin-1 mRNA in the JTE-013 group was higher than that in the SAP group, and the differences were statistically significant (**Figure 5C**, *P<0.05*).

### 3.5 S1PR2 facilitated pyroptosis of THP-1 *in vitro*

**Figure 6:**
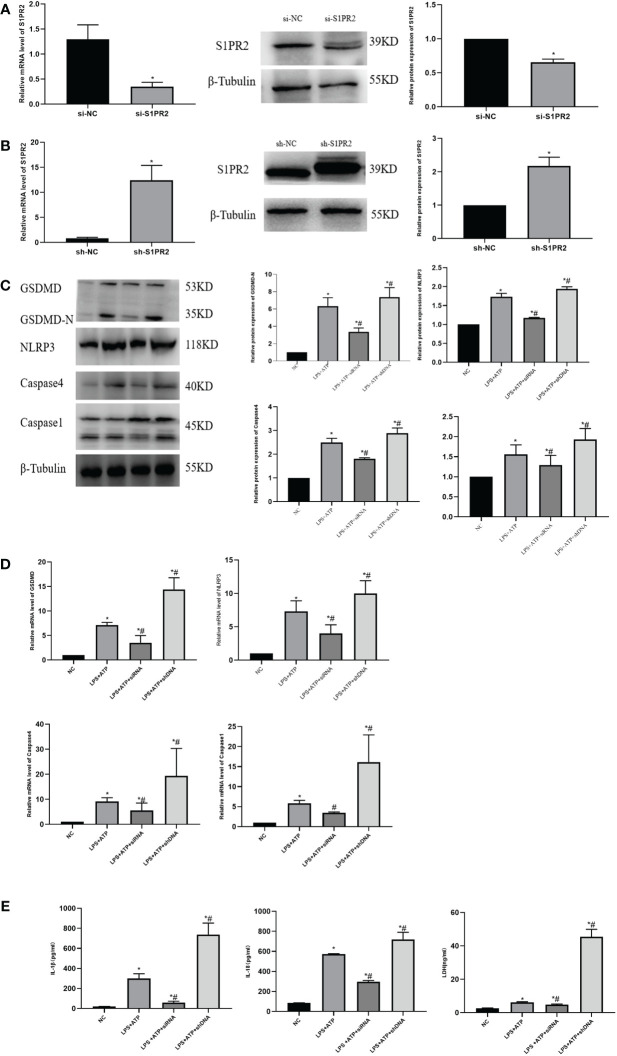
The pyroptosis level of THP-1 cells was detected *in vitro* **(A)** The expression of S1PR2 protein transfected siRNA detected by western blot and the mRNA level detected by PCR. **(B)** The expression of S1PR2 protein transfected shDNA detected by western blot and the mRNA level detected by PCR. **(C)** The expression of pyroptosis protein was detected by western blot *in vitro*. **(D)** The mRNA level of pyroptosis in THP-1 detected by PC^©^ **(E)** The level of IL-18、IL-1β、LDH in THP-1 supernatant detected by ELISA. *P<0.05 VS.NC #P<0.05 VS.LPS+ATP.

We first transfected cells with siRNA or overexpression plasmids for 48 h, respectively, and detected the S1PR2 protein expression by Western Blot (**Figures 6A, B**). To verify the role of S1PR2 in pyroptosis, we assessed pyroptosis-related proteins in THP-1 groups, and the results showed that the expressions of pyroptosis proteins GSDMD-N, NLRP3, Caspase1 and Caspase4 in the LPS+ATP+shDNA group were higher than those in the LPS+ATP group. Compared with the LPS+ATP group, the pyroptosis related proteins in the LPS+ATP+siRNA group decreased significantly (**Figure 6C**), indicating that S1PR2 is correlated with pyroptosis. The PCR results similarly demonstrated statistically significant disparities in mRNA levels among the groups (**Figure 6D**). Additionally, we assessed the levels of inflammatory biomarkers, including LDH, IL-18, and IL-1β, following pyroptosis induction. The results revealed statistically significant variations among groups (**Figure 6E**).

### 3.6 The RhoA/ROCK pathway induces pyroptosis in THP-1 cells, causing cellular damage to FHC

**Figure 7:**
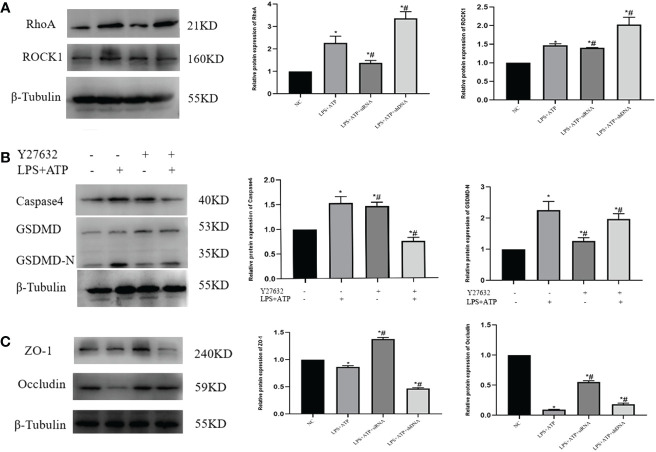
Y27632 inhibits the RhoA/ROCK pathway *in vitro* **(A)** LPS+ATP activated the expression of RhoA/ROCK1 *in vitro* detected by western blot. **(B)** The necessity of The RhoA/ROCK pathway and pyroptosis was verified by the rescue experiment. **(C)** Expression of adhesion protein after co-culture of FHC and THP-1 in each group. *P<0.05 VS.NC #P<0.05 VS.LPS+ATP.

The expression levels of RhoA/ROCK were initially assessed in each cell group, revealing a statistically significant disparity (**Figure 7A**). To further investigate the relationship between the RhoA/ROCK pathway and macrophage pyroptosis, as well as to determine whether S1PR2 regulates this pathway, we treated THP-1 cells overexpressing S1PR2 with the RhoA/ROCK pathway inhibitor Y27632 in order to assess changes in pyroptosis-related proteins. The results demonstrated a significant decrease in the expression levels of GSDMD-N, Caspase4, and associated proteins following Y27632 treatment (**Figure 7B**).

We further investigated the impact of macrophage-induced pyroptosis on intestinal epithelial cells by co-culturing THP-1 and FHC cells in small chambers, followed by the detection of ZO-1 and Occludin-related proteins. The results revealed a significant decrease in the expression of ZO-1 and Occludin in cells from the LPS+ATP+shDNA group compared to the control group. Conversely, when comparing with the LPS+ATP group, there was a notable increase in ZO-1 and Occludin expression in the LPS+ATP+siRNA group (**Figure 7C**), indicating that macrophage pyroptosis had an impact on tight junction protein expression within intestinal epithelial cells.

## 4 Discussion

Sphingosine-1-phosphate (S1P) is a bioactive lipid molecule that regulates various cellular functions through its binding to the S1PR receptor in different cell types. S1PR2, the most highly expressed S1P receptor on macrophages’ surface, interacts with extracellular factors to mediate diverse reactions. Previous studies have shown that knocking down S1PR2 in the liver can alleviate chronic glucocorticoid-induced glucose intolerance and reduce fasting plasma insulin level (12). S1P plays an important role in the cardiovascular system by participating in the occurrence and development of diseases related to the cardiovascular system (13). Previous studies have shown that combined S1P with S1PR2 can regulate the NLRP3 inflammasome and promote fibrosis in chronic pancreatitis (14). The S1PR2 signaling pathway is associated with the metastasis process of pancreatic cancer (15). In addition, S1PR2 is up-regulated in mice with cholestatic liver fibrosis, while JTE-013 can reduce liver histopathological injury, collagen accumulation and fibrosis-related gene expression in mice (16). Furthermore, the researchers found that derivatives of JTE-013 exhibit promising potential as novel and effective antagonists targeting S1PR2 for colorectal cancer treatment (17). Our study revealed upregulation of intestinal expression of S1RP2 in mice with SAP, moreover, when we inhibit this protein by JTE-013, we found that the degree of pancreatic and intestinal injury decreased. These findings suggest that the involvement of S1PR2 may contribute to the pathogenesis and progression of SAP, thereby it may be a potential therapeutic mechanism for utilizing JTE-013 in the treatment of SAP with intestinal disorders.

Pyroptosis, accompanied by the cleavage of GSDMD and the release of the associated inflammatory cytokines IL-18 and IL-1β, is mediated by the assembly of inflammasome (8). Pyroptosis is involved in the occurrence and development of many diseases in the body, including cardiovascular system diseases (18), nervous system diseases (19), cancer and other diseases (20). Mechanistically, pyroptosis can be classified into two main pathways: a classical pathway involving activation of Caspase1-dependent inflammasomes like NLRP3; a non-classical pathway mediated by Caspase 4/11. In these signaling pathways, GSDMD undergoes enzymatic processing by respective Caspases to generate an active N-terminal fragment that self-assembles into pore-forming structures on cellular membranes, leading to the efflux of intracellular contents and ultimately resulting in pyroptosis (21).

The study showed that LPS can upregulate the expression of Caspase11 (22), induce non-classical pathway pyroptosis of cells, and cause disruption of the cell membrane to release IL-18 and IL-1β (23). Previous studies have demonstrated an increase in intestinal bacterial colonization following down-regulation of Caspase4/11 expression. Intracellular (Salmonella typhimurium) and extracellular (enteropathogenic Escherichia coli) intestinal pathogens can activate the Caspase-4 inflammasome, promoting the activation of pro-inflammatory cytokine IL-18 via intracellular LPS (24). In our study, we observed that the induction of THP-1 with a combination of LPS and ATP resulted in pyroptosis *in vitro*, accompanied by an upregulation of pyroptosis-related proteins and increased levels of IL-18 in the cell supernatant. However, when we knockdown the expression of S1PR2, we observed a decrease in the level of pyroptosis in THP-1 cells. These findings suggest that S1PR2 may serve as a potential promoter for pyroptosis. Furthermore, we observed that overexpression of S1PR2 led to an enhanced level of pyroptosis in THP-1, emphasizing this protein played a crucial role in regulating pyroptosis. Previous studies have shown that the RhoA/ROCK pathway plays an important role in neurodegenerative diseases and cardiovascular diseases (25, 26), and inhibiting the RhoA/ROCK pathway can reduce the progression of Parkinson’s disease (27). At the same time, the RhoA/ROCK pathway is involved in the development of ovarian cancer (28). In our study, we have demonstrated the involvement of the RhoA/ROCK pathway in pyroptosis by its inhibition, which not only reduces pyroptosis but also mitigates inflammation. Our findings highlight the impact of the RhoA/ROCK pathway on pyroptosis and suggest that targeting this pathway could serve as a novel therapeutic approach for SAP treatment.

The intestinal barrier includes mechanical barrier, chemical barrier, immune barrier, and biological barrier (29). The integrity of the intestinal mechanical barrier is mainly supported by the tight connection of epithelial cells, which effectively prevents harmful substances (such as pathogens and endotoxins) from entering the blood through the intestinal mucosa. It is a key cellular process to maintain the stability of the intestinal epithelium and tight junctions, and the permeability of intestine may increase accompanied by the downregulation of tight junctions (30). The extent of intestinal damage in SAP is crucial for the prognosis. Previous studies have shown that there are three distinct types of intercellular junctions within intestinal cells: tight junctions, adhesive junctions, and desmosomes (31). The apical junction complex, composed of multiple proteins, collectively supports the formation of dense microvilli brush boundaries and plays a crucial role in regulating epithelial barrier function and intercellular transport. Among these proteins, ZO-1 is particularly essential for maintaining the permeability of epithelial cells (32). Although the ultrastructure and function of the epithelial barrier have been extensively characterized, there is currently no proven pharmacotherapy available. It has not been demonstrated that restoring barrier function can improve the clinical presentation of local gastrointestinal diseases or systemic diseases effectively, and the intestinal barrier function still has some potential for clinical treatment (33). In our study, the expression of intestinal adhesion proteins was utilized as an indicator to evaluate enterocyte functionality. Through co-culturing THP-1 and FHC, we observed that induction of macrophage pyroptosis led to varying degrees of impairment in FHC, leading to a reduction in the expression of surface cell adhesion proteins. Additionally, the inhibition of S1PR2 expression in macrophages resulted in a reduction of FHC function. Consequently, the occurrence of THP-1 pyroptosis decreased along with a corresponding reduction in the extent of FHC injury, indicating that S1PR2 may be a potential therapeutic target for treating intestinal injury induced by SAP.

## 5 Conclusion

In conclusion, our study suggests that the involvement of S1PR2 in severe acute pancreatitis-induced intestinal mucosal barrier dysfunction is mediated by its regulation of macrophage pyroptosis via the RhoA/ROCK pathway.

## Data availability statement

The original contributions presented in the study are included in the article/supplementary material. Further inquiries can be directed to the corresponding author.

## Ethics statement

The animal study was approved by the Affiliated Hospital of Qingdao University Animal Care and Welfare Committee. The study was conducted in accordance with the local legislation and institutional requirements.

## Author contributions

TL: Formal analysis, Writing – original draft, Writing – review & editing. MP: Formal analysis, Writing – original draft. QZ: Formal analysis, Writing – review & editing. XP: Funding acquisition, Supervision, Writing – review & editing.

## References

[1] Hines OJ Pandol SJ. Management of severe acute pancreatitis. BMJ. (2019) 367:l6227. doi: 10.1136/bmj.l6227 31791953

[2] Ge P Luo Y Okoye CS Chen H Liu J Zhang G. Intestinal barrier damage, systemic inflammatory response syndrome, and acute lung injury: A troublesome trio for acute pancreatitis. BioMed Pharmacother. (2020) 132:110770. doi: 10.1016/j.biopha.2020.110770 33011613

[3] Maurer LR Fagenholz PJ. Contemporary surgical management of pancreatic necrosis. JAMA Surg. (2023) 158:81–8. doi: 10.1001/jamasurg.2022.5695 36383374

[4] Kwong E Li Y Hylemon PB Zhou H. Bile acids and sphingosine-1-phosphate receptor 2 in hepatic lipid metabolism. Acta Pharm Sin B. (2015) 5:151–7. doi: 10.1016/j.apsb.2014.12.009 PMC4629213 26579441

[5] Chen H Wang J Zhang C Ding P Tian S Chen J. Sphingosine 1-phosphate receptor, a new therapeutic direction in different diseases. BioMed Pharmacother. (2022) 153:113341. doi: 10.1016/j.biopha.2022.113341 35785704

[6] Shi J Zhao Y Wang K Shi X Wang Y Huang H. Cleavage of GSDMD by inflammatory caspases determines pyroptosis cell death. Nature. (2015) 526:660–5. doi: 10.1038/nature15514 26375003

[7] Coll RC Schroder K Pelegrín P. NLRP3 and pyroptosis blockers for treating inflammatory diseases. Trends Pharmacol Sci. (2022) 43:653–68. doi: 10.1016/j.tips.2022.04.003 35513901

[8] Yu P Zhang X Liu N Tang L Peng C Chen X. Pyroptosis: mechanisms and diseases. Signal Transduct Target Ther. (2021) 6:128. doi: 10.1038/s41392-021-00507-5 33776057 PMC8005494

[9] Song F Hou J Chen Z Cheng B Lei R Cui P. Sphingosine-1-phosphate receptor 2 signaling promotes caspase-11-dependent macrophage pyroptosis and worsens escherichia coli sepsis outcome. Anesthesiology. (2018) 129:311–20. doi: 10.1097/ALN.0000000000002196 29620575

[10] Schmidt J Rattner DW Lewandrowski K Compton CC Mandavilli U Knoefel WT. A better model of acute pancreatitis for evaluating therapy. Ann Surg. (1992) 215:44–56. doi: 10.1097/00000658-199201000-00007 1731649 PMC1242369

[11] Chiu CJ Scott HJ Gurd FN. Intestinal mucosal lesion in low-flow states. II. The protective effect of intraluminal glucose as energy substrate. Arch Surg. (1970) 101:484–8. doi: 10.1001/archsurg.1970.01340280036010 5311679

[12] Lee RA Chang M Tsay A Lee YR Li D Yiv N. Chronic glucocorticoid exposure induced a S1PR2-RORγ Axis to enhance hepatic gluconeogenesis in male mice. Diabetes. (2023) 11:1534–46. doi: 10.2337/db22-0605 PMC10588286 37552863

[13] Wang N Li JY Zeng B Chen GL. Sphingosine-1-phosphate signaling in cardiovascular diseases. Biomolecules. (2023) 13:818. doi: 10.3390/biom13050818 37238688 PMC10216071

[14] Cui L Li C Zhang G Zhang L Yao G Zhuo Y. S1P/S1PR2 promote pancreatic stellate cell activation and pancreatic fibrosis in chronic pancreatitis by regulating autophagy and the NLRP3 inflammasome. Chem Biol Interact. (2023) 380:110541. doi: 10.1016/j.cbi.2023.110541 37169277

[15] Sarkar J Aoki H Wu R Aoki M Hylemon P Zhou H. ASO visual abstract: conjugated bile acids accelerate progression of pancreatic cancer metastasis via S1PR2 signaling in cholestasis. Ann Surg Oncol. (2023) 30:1644–5. doi: 10.1245/s10434-022-12915-0 PMC9911402 36396870

[16] Yang J Tang X Liang Z Chen M Sun L. Taurocholic acid promotes hepatic stellate cell activation via S1PR2/p38 MAPK/YAP signaling under cholestatic conditions. Clin Mol Hepatol. (2023) 29:465–81. doi: 10.3350/cmh.2022.0327 PMC10121313 36800698

[17] Guo Z Zhang S Liu X Zhao G Zhang Y Luo D. Design, synthesis, and evaluation of JTE-013 derivatives as novel potent S1PR2 antagonists for recovering the sensitivity of colorectal cancer to 5-fluorouracil. Bioorg Chem. (2023) 131:106318. doi: 10.1016/j.bioorg.2022.106318 36527992

[18] Zhaolin Z Guohua L Shiyuan W Zuo W. Role of pyroptosis in cardiovascular disease. Cell Prolif. (2019) 52:e12563. doi: 10.1111/cpr.12563 30525268 PMC6496801

[19] Li S Sun Y Song M Song Y Fang Y Zhang Q. NLRP3/caspase-1/GSDMD-mediated pyroptosis exerts a crucial role in astrocyte pathological injury in mouse model of depression. JCI Insight. (2021) 6:e146852. doi: 10.1172/jci.insight.146852 34877938 PMC8675200

[20] Loveless R Bloomquist R Teng Y. Pyroptosis at the forefront of anticancer immunity. J Exp Clin Cancer Res. (2021) 40:264. doi: 10.1186/s13046-021-02065-8 34429144 PMC8383365

[21] Fang Y Tian S Pan Y Li W Wang Q Tang Y. Pyroptosis: A new frontier in cancer. Biomedicine Pharmacotherapy. (2020) 121:109595. doi: 10.1016/j.biopha.2019.109595 31710896

[22] Wang S Miura M Jung YK Zhu H Li E Yuan J. Murine caspase-11, an ICE-interacting protease, is essential for the activation of ICE. Cell. (1998) 92:501–9. doi: 10.1016/s0092-8674(00)80943-5 9491891

[23] Shi J Gao W Shao F. Pyroptosis: gasdermin-mediated programmed necrotic cell death. Trends Biochem Sci. (2017) 42:245–54. doi: 10.1016/j.tibs.2016.10.004 27932073

[24] Knodler LA Crowley SM Sham HP Yang H Wrande M Ma C. Noncanonical inflammasome activation of caspase-4/caspase-11 mediates epithelial defenses against enteric bacterial pathogens. Cell Host Microbe. (2014) 16:249–56. doi: 10.1016/j.chom.2014.07.002 PMC4157630 25121752

[25] Schmidt SI Blaabjerg M Freude K Meyer M. RhoA signaling in neurodegenerative diseases. Cells. (2022) 11:1520. doi: 10.3390/cells11091520 35563826 PMC9103838

[26] Seccia TM Rigato M Ravarotto V Calò LA. ROCK (RhoA/rho kinase) in cardiovascular-renal pathophysiology: A review of new advancements. J Clin Med. (2020) 9:1328. doi: 10.3390/jcm9051328 32370294 PMC7290501

[27] Iyer M Subramaniam MD Venkatesan D Cho SG Ryding M Meyer M. Role of RhoA-ROCK signaling in Parkinson’s disease. Eur J Pharmacol. (2021) 894:173815. doi: 10.1016/j.ejphar.2020.173815 33345850

[28] Wei X Lou H Zhou D Jia Y Li H Huang Q. TAGLN mediated stiffness-regulated ovarian cancer progression via RhoA/ROCK pathway. J Exp Clin Cancer Res. (2021) 40:292. doi: 10.1186/s13046-021-02091-6 34538264 PMC8451140

[29] Albillos A de Gottardi A Rescigno M. The gut-liver axis in liver disease: Pathophysiological basis for therapy. J Hepatol. (2020) 72:558–77. doi: 10.1016/j.jhep.2019.10.003 31622696

[30] Dong L Xie J Wang Y Jiang H Chen K Li D. Mannose ameliorates experimental colitis by protecting intestinal barrier integrity. Nat Commun. (2022) 13:4804. doi: 10.1038/s41467-022-32505-8 35974017 PMC9381535

[31] Parigi SM Larsson L Das S Ramirez Flores RO Frede A Tripathi KP. The spatial transcriptomic landscape of the healing mouse intestine following damage. Nat Commun. (2022) 13:828. doi: 10.1038/s41467-022-28497-0 35149721 PMC8837647

[32] Kuo WT Zuo L Odenwald MA Madha S Singh G Gurniak CB. The tight junction protein ZO-1 is dispensable for barrier function but critical for effective mucosal repair. Gastroenterology. (2021) 161:1924–39. doi: 10.1053/j.gastro.2021.08.047 PMC8605999 34478742

[33] Camilleri M. The leaky gut: mechanisms, measurement and clinical implications in humans. Gut. (2019) 68:1516–26. doi: 10.1136/gutjnl-2019-318427 PMC6790068 31076401

